# Surgical site infection prevention: a scoping review of strategies, implementation barriers, and emerging approaches

**DOI:** 10.3389/fpubh.2026.1884446

**Published:** 2026-07-30

**Authors:** Sara Yousef Falah Abualkishik, Noorsuzana Mohd Shariff, Rohayu Binti Hami

**Affiliations:** 1 Department of Community Health, Pusat Kanser Tun Abdullah Ahmad Badawi, Universiti Sains Malaysia, Kepala Batas, Penang, Malaysia; 2 Department of Nursing, Faculty of Nursing, Irbid National University, Irbid, Jordan

**Keywords:** antibiotic prophylaxis, implementation challenges, low- and middle-income countries, negative pressure wound therapy, scoping review, SSI prevention, surgical site infections

## Abstract

**Background:**

Surgical site infections (SSIs) remain a major cause of healthcare-associated morbidity, mortality, and increased healthcare costs worldwide, particularly in low- and middle-income countries (LMICs). Although evidence-based prevention guidelines are widely available, inconsistent implementation and variability in adherence continue to limit their effectiveness across healthcare settings.

**Methods:**

This scoping review synthesized recent evidence on SSI prevention strategies published between 2019 and 2025. A systematic search of Scopus, Web of Science, and ScienceDirect identified 24 eligible studies. Data were extracted and thematically analyzed to examine intervention effectiveness, implementation challenges, and emerging innovations in SSI prevention.

**Results:**

Core clinical strategies—including antibiotic prophylaxis, topical antisepsis and decolonization, and negative pressure wound therapy (NPWT)—were consistently effective; however, their success depended heavily on implementation fidelity. Multicomponent strategies, particularly prevention bundles, significantly improved outcomes by enhancing consistency and reducing variability in care delivery. Persistent barriers, including limited resources, inadequate training, and workflow constraints, remained major obstacles across both high- and low-resource settings. In LMICs, context-specific and low-cost interventions, such as glove and instrument changes before wound closure, demonstrated substantial effectiveness and scalability. Emerging data-driven approaches, including artificial intelligence and machine learning, showed strong potential in improving SSI risk prediction and surveillance, although infrastructural limitations continue to restrict broader implementation.

**Conclusion:**

SSI prevention is fundamentally an implementation-driven challenge requiring alignment between evidence-based interventions, system-level capacity, and context-sensitive adaptation. By integrating clinical, implementation, and technological perspectives, this review provides a unified framework to guide future research, policy development, and healthcare practice aimed at reducing the global burden of SSIs.

## 1 Introduction

Surgical site infections (SSIs) are infections occurring at or near a surgical incision within 30 days of a procedure, or up to 1 year when an implant is involved. They are among the most common and preventable healthcare-associated infections (HAIs), yet continue to pose a significant global health challenge. Despite advancements in surgical techniques, antimicrobial prophylaxis, and aseptic practices, SSIs remain a persistent burden on healthcare systems, contributing to adverse patient outcomes and increased healthcare costs (1, 2). Globally, SSIs are associated with increased morbidity and mortality, prolonged hospitalization, and substantial financial strain. Complications such as delayed wound healing, secondary infections, and sepsis further exacerbate their impact. This burden is disproportionately higher in low- and middle-income countries (LMICs), where limited resources and inconsistent infection control practices intensify the problem (3). SSI rates have been reported to reach up to 11% among surgical patients in LMICs and up to 20% in procedures such as caesarean sections in Africa (4), underscoring the need for context-sensitive prevention strategies.

SSI prevention encompasses interventions across the preoperative, intraoperative, and postoperative phases, including antibiotic prophylaxis, skin antisepsis, sterile surgical techniques, wound care, and environmental controls (5). Substantial evidence supports the effectiveness of these measures. For instance, timely antibiotic administration significantly reduces SSI risk (6), while alcohol-based chlorhexidine has demonstrated superiority over iodine-based preparations in reducing surgical wound contamination (7). However, the effectiveness of these interventions is highly dependent on consistent adherence to established protocols. In practice, adherence remains variable across healthcare settings, particularly in resource-constrained environments. Persistent gaps in antibiotic stewardship, sterile technique, and postoperative wound care continue to contribute to preventable infections (8), with non-compliance recognized as a major global barrier to effective SSI prevention (9).

The economic and societal burden of SSIs is substantial. In high-income settings, SSIs can add significant costs per patient due to prolonged hospitalization, readmissions, additional surgical interventions, and increased antimicrobial use (5). In LMICs, these financial pressures are even more critical, often diverting limited healthcare resources from essential services (2). Beyond economic impact, SSIs significantly affect patient quality of life, leading to prolonged recovery, reduced physical functioning, and psychological distress (10). Vulnerable populations, including the older adults and immunocompromised individuals, face higher risks, and delays in diagnosis and treatment in resource-limited settings may lead to severe or fatal outcomes (3).

Despite global progress in infection prevention and control (IPC), several systemic challenges remain unresolved. Variability in definitions and surveillance systems limits comparability of SSI data across settings (11), while infrastructural and resource limitations continue to constrain effective implementation, particularly in LMICs (12). Although cost-effective interventions—such as glove and instrument changes before wound closure—have demonstrated effectiveness (13), their adoption remains inconsistent due to logistical, financial, and organizational barriers.

In parallel, advances in prevention strategies, including multicomponent care bundles and emerging data-driven approaches such as artificial intelligence (AI)-based risk prediction and surveillance systems, have introduced new opportunities for improving SSI outcomes. However, the comparative effectiveness of these innovations and their integration into routine clinical practice remain insufficiently explored, particularly across diverse healthcare contexts (14, 15).

Given these persistent gaps, there is a need for a comprehensive synthesis of recent evidence that not only identifies effective SSI prevention strategies but also examines the contextual and implementation factors influencing their success. This scoping review therefore synthesizes peer-reviewed studies published between 2019 and 2025, with the aim of informing clinical practice, policy development, and future research.

The review is guided by the following research questions:

- What evidence-based strategies have been implemented to prevent SSIs in healthcare settings between 2019 and 2025?
- What are the key challenges and innovations associated with the implementation of SSI prevention strategies?

## 2 Method

### 2.1 Search strategy

A comprehensive literature search was conducted in Scopus, Web of Science, and ScienceDirect to identify studies on surgical site infection (SSI) prevention. The selection of these sources was guided by the mapping purpose of the scoping review and by the need to capture evidence across clinical, surgical, nursing, infection prevention, public health, and implementation-related literature. Scopus and Web of Science were used as major multidisciplinary citation databases because they support broad retrieval across health and non-health disciplines and allow identification of literature that may sit at the intersection of clinical practice, health systems, and implementation research. ScienceDirect was searched as a complementary full-text source because of its coverage of peer-reviewed clinical, surgical, nursing, and health sciences journals. Together, these sources were considered appropriate for mapping the breadth of recent SSI prevention evidence across clinical interventions, implementation challenges, and context-specific healthcare practices. The search targeted English-language articles published from January 2019 to December 2025 to reflect recent advancements. The 2019 start date was deliberately chosen to capture evidence following the publication of major global guidelines: the WHO Global Guidelines for the Prevention of Surgical Site Infection (2018) and the CDC Guideline for the Prevention of Surgical Site Infection (2017).

The search strategy was developed using an iterative approach, guided by the Population–Concept–Context (PCC) framework recommended for scoping reviews.

The population included surgical patients and healthcare professionals, the concept focused on SSI prevention strategies, and the context encompassed healthcare settings such as hospitals, surgical units, and operating rooms. Based on these elements, key search terms surgical site infection, infection prevention, and infection control were identified and refined through preliminary searches.

Search terms were adapted for each database to account for differences in indexing and functionality, and were combined using Boolean operators (AND, OR) to maximize sensitivity and relevance. The full database-specific search strings, including Boolean operators and applied filters, are provided in Supplementary File A. The search process was iterative, allowing refinement of keywords to reduce irrelevant results while maintaining comprehensive coverage, consistent with recommended scoping review methodology. The search was limited to articles published in English between January 2019 and December 2025.

### 2.2 Study selection process

The study selection process followed the PRISMA-ScR guidelines (16) to ensure transparency and methodological rigor. A total of 11.635 records were initially retrieved and uploaded into Rayyan, a web-based systematic review tool (17). After removing 2,785 duplicates, 8850 unique records remained.

Two reviewers (SYFA and NMS) independently screened titles and abstracts. A total of 8,632 articles were excluded for the following reasons: irrelevance to SSI prevention (*n* = 5,656), focus on non-healthcare settings (*n* = 1,493), publication type (e.g., opinion pieces or editorials) (*n* = 653), and abstract-only publications (*n* = 830). Two hundred and eighteen full-text articles were subsequently assessed for eligibility.

Of these, 192 studies were excluded due to the following reasons: focus on SSI infections is limited (*n* = 69), evaluation of unrelated interventions (*n* = 86), and methodological limitations such as insufficient reporting or incomplete data (*n* = 39). Ultimately, 24 studies met the inclusion criteria and were included in the final synthesis.

**Figure 1:**
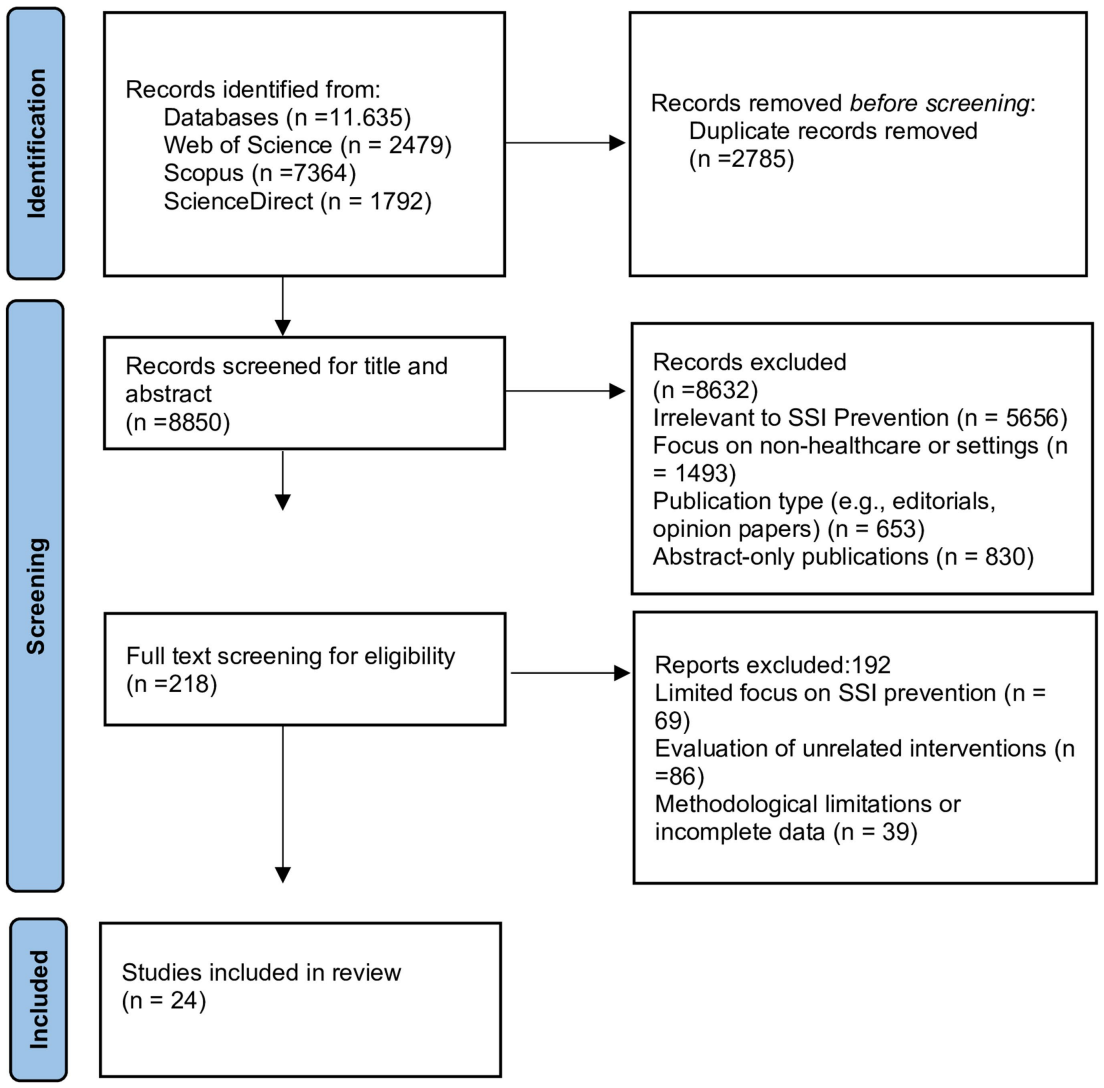
PRISMA flow diagram illustrating the study selection process for the scoping review. Adapted from the PRISMA (36) flow diagram template and populated with the data from this review. Creative Commons Attribution 4.0 International (CC BY 4.0).

Screening was conducted independently by both reviewers, and discrepancies were resolved through discussion and consensus. A third reviewer was consulted where necessary to resolve disagreements. The study selection process is summarized in Figure 1 (PRISMA Flow Diagram).

### 2.3 Inclusion and exclusion criteria

Studies were included based on the following criteria:

1. Population: Surgical patients or healthcare professionals involved in SSI prevention.
2. Concept: Studies describing or evaluating SSI prevention strategies, including antibiotic prophylaxis, antisepsis, wound care, sterile techniques, patient education, and environmental controls.
3. Context: Clinical settings such as hospitals, surgical wards, and outpatient surgical units.
4. Study Types: Original empirical studies (e.g., randomized controlled trials, cohort studies, case–control studies, and cross-sectional studies) and evidence-based guidelines or policies from recognized organizations (e.g., WHO, CDC).

Studies were excluded if they did not focus on SSIs, were review articles, meta-analyses, opinion pieces, or abstracts without full text, were published in languages other than English, fell outside the 2019–2025 timeframe, or were conducted in non-clinical or non-surgical settings. These criteria ensured alignment with the review objectives and relevance to SSI prevention practices.

### 2.4 Characteristics of included studies

Data from each included study were systematically documented using a standardized extraction framework. Extracted information included study title, authors, year of publication, study design (e.g., randomized controlled trials, cohort, or case–control studies), geographical location, healthcare setting, population characteristics, sample size, study objectives, key findings, and relevance to SSI prevention.

In addition, each study’s strengths and limitations were recorded to provide contextual understanding of the evidence base and to support interpretation of findings.

### 2.5 Critical appraisal

Methodological quality of the included studies was assessed using the Mixed Methods Appraisal Tool (MMAT) (18). The appraisal focused on criteria such as clarity of research questions, appropriateness of study design, data collection methods, and rigor of analysis. As MMAT is designed for empirical qualitative, quantitative, and mixed-methods studies, expert consensus and guideline-based sources were not appraised using MMAT. These sources were retained as contextual evidence to support interpretation of SSI prevention practices, guideline recommendations, and implementation considerations.

Of the 24 included studies, 75% met at least 80% of the MMAT criteria, indicating strong methodological quality. The remaining 25% demonstrated limitations such as incomplete reporting, retrospective designs, or potential bias, which may affect the generalizability of findings.

MMAT appraisal was conducted independently by two reviewers, and discrepancies were resolved through discussion. The MMAT results were used descriptively to indicate study quality and were not used as a basis for exclusion. Detailed appraisal results are presented in Table 1.

**Table 1:** MMAT appraisal.

**#**	**Study details**	**Study type**	**Criteria 1**	**Criteria 2**	**Criteria 3**	**Criteria 4**	**Criteria 5**	**Comments**
1	Evaluating antibiotic prophylaxis adherence: Implications for surgical site infections and wound care management	Quantitative	Yes	Yes	Yes	No	Yes	High loss to follow-up (21.3%) could introduce bias. Representativeness unclear.
2	Routine sterile glove and instrument change at the time of abdominal wound closure to prevent surgical site infection	Quantitative	Yes	Yes	Yes	Yes	Yes	The study uses a decision-analytic model, includes a large sample from multiple countries, and has clear, precise results. Minimal cost difference and slight reduction in SSI rates.
3	Implementing a surgical site infection prevention bundle for emergency appendectomy	Quantitative	Yes	Yes	Yes	Yes	Yes	The study evaluates the feasibility and impact of an intraoperative SSI prevention bundle and shows significant reduction in SSI rates. The statistical analysis (multinomial logistic regression) is appropriate. High compliance to the bundle (79.9%) and a decrease in SSIs post-implementation support the intervention’s effectiveness.
4	Implementation of a surgical site infection prevention bundle in gynecologic oncology patients: An enhanced recovery after surgery initiative	Quantitative	Yes	Yes	Yes	Yes	Yes	Study clearly addresses the research question with strong statistical methods and an appropriate sample. The retrospective nature may introduce bias but is mitigated by statistical adjustments.
5	Impact of control measures including decolonization and hand hygiene for orthopedic surgical site infection caused by MRSA at a Japanese tertiary-care hospital	Quantitative	Yes	Yes	Yes,	Yes	Yes	The study uses strong epidemiological and molecular methods. The retrospective nature and lack of randomization may introduce biases but are mitigated by robust control measures and data collection.
6	Preventive strategies in pediatric cardiovascular surgery: impact on surgical site infections and beyond	Quantitative	Yes	Yes	Yes	Yes	Yes	The study’s strengths include a large sample size (2,512 surgeries), use of statistical models, and focus on relevant interventions. However, the observational nature and lack of randomization are potential limitations.
7	Negative pressure wound therapy reduces surgical site infections	Quantitative	Yes	Yes	Yes	Yes	Yes	The study’s strengths include a relatively large sample size and the use of regression analysis. However, the lack of randomization could introduce bias.
8	Appropriateness of choice and duration of surgical antibiotic prophylaxis and the incidence of surgical site infections	Quantitative	Yes	Yes	Yes	Yes	Yes	The study’s strengths include clear inclusion criteria and a well-defined population. The lack of control for potential confounders (e.g., patient characteristics) is a limitation.
9	Topical use of hyperoxygenated fatty acids decreases surgical site infection in patients following laparoscopic cholecystectomy	Quantitative (RCT)	Yes	Yes	Yes	Yes	Yes	The study is well-structured and robust. The key limitation is that it only focuses on one surgical procedure (laparoscopic cholecystectomy) and one specific treatment (HOFA), limiting the generalizability to other settings or surgeries
10	Individual decontamination measures reduce by two the incidence of surgical site infections in spinal surgery	Quantitative	Yes	Yes	Yes	Yes	Yes	The study provides strong evidence for the effectiveness of the decolonization protocol. The limitation is the observational design without a true control group, which may weaken the ability to establish causality.
11	Role of antibiotic prophylaxis on surgical site infection prevention in a low-risk population undergoing laparoscopic cholecystectomy	Quantitative (RCT)	Yes	Yes	Yes	Yes	Yes	The RCT design strengthens the internal validity. However, the study’s small sample size and lack of significant findings limit the generalizability of the results.
12	Improving surgical site infection prevention in Asia-Pacific through appropriate surveillance programs: Challenges and recommendations	Expert panel discussion (Qualitative review)	Yes	Yes	Yes	No	No	The study provides valuable insights from experts on SSI prevention but lacks empirical data and statistical analysis. Its qualitative nature limits the ability to generalize findings to larger populations.
13	Task-based training to prevent surgical site infection: A formative evaluation	Mixed-Methods	Yes	Yes	Yes	No	Yes	The study provides useful insights into how task-based training can improve infection prevention practices in an LMIC setting. The main limitation is the lack of objective measures to assess the long-term impact on infection prevention practices.
14	Development and validation of a point-of-care clinical risk score to predict surgical site infection following open spinal fusion	Quantitative	Yes	Yes	Yes	Yes	Yes	The study provides valuable insights into predictive analytics for SSI risk, with a high degree of statistical validation. Limitations include its retrospective nature and lack of prospective validation in clinical settings.
15	Impact of postoperative skin disinfection with chlorhexidine on bacterial colonization following shoulder arthroplasty surgery: a controlled randomized study	Quantitative (RCT)	Yes	Yes	Yes	Yes	Yes	The study design is solid and adheres to RCT standards, though the lack of significant findings limits its impact. Further research with different methods or larger samples may be needed.
16	Construction and validation of nomogram to predict surgical site infection after hysterectomy: a retrospective study	Quantitative	Yes	Yes	Yes	Yes	Yes	The study design is well-executed with appropriate analyses and a practical tool (nomogram). However, its retrospective nature and single-center setting may limit the broader application of findings. Further prospective studies could strengthen the conclusions.
17	Development of machine learning models for the detection of surgical site infections following total hip and knee arthroplasty: a multicenter cohort study	Quantitative	Yes	Yes	Yes	Yes	Yes	The study provides valuable insights into machine learning’s role in SSI surveillance. While robust, future research could address potential biases in administrative data and validate findings across different healthcare settings.
18	Strategies to Prevent Surgical Site Infections in Acute-Care Hospitals: 2022 Update	Expert Guidelines Update	Yes	Yes	Yes	Yes	Yes	The study is comprehensive and aligns with expert guideline standards. However, its applicability to non-acute care or resource-limited settings may be limited.
19	Intervention Strategies to Reduce Surgical Site Infection Rates in Patients Undergoing Rectal Cancer Surgery	Quantitative	Yes	Yes	Yes	Yes	Yes	The study effectively demonstrates a reduction in SSI rates over time. However, the retrospective design limits the ability to establish causation. Further prospective studies are needed to confirm the effectiveness of specific interventions.
20	Effectiveness of Prophylactic Intranasal Photodynamic Disinfection Therapy and Chlorhexidine Gluconate Body Wipes for SSI Prophylaxis in Adult Spine Surgery	Quantitative	Yes	Yes	Yes	Yes	Yes	The study effectively shows the intervention’s clinical and financial benefits. However, other practice changes and variability in data reporting are not fully accounted for.
21	Wound care practices across two acute care settings: A comparative study	Quantitative	Yes.	Yes	Yes	Yes	Yes	The study effectively compares wound care practices across two hospitals, but limitations include the potential Hawthorne effect and site-specific contextual factors.
22	Sigari and Abdolhoseinpour (28) “Operative site irrigation with povidone-iodine solution in spinal surgery for SSI prevention”	Quantitative	Yes	Yes	Yes	Yes	Yes	A well-designed RCT, but single-center data and limited external validation constrain generalizability.
23	Machine Learning-Enhanced Surveillance for Surgical Site Infections in Patients Undergoing Colon Surgery	Quantitative	Yes	Yes	Yes	Yes	Yes	Strong methodology; single-center design and class imbalance (4.4% SSI) are key limitations. Requires multi-site external validation.
24	Evaluating the Performance of State-of-the-Art Artificial Intelligence Chat Bots Based on the WHO Global Guidelines for the Prevention of Surgical Site Infection	Quantitative	Yes	Yes	Yes	Yes	Yes	Validated QUEST framework used; blinded expert evaluation. Limitations: cross-sectional design, first-response only, closed-source models.

### 2.6 Data extraction and synthesis

Data were extracted using a standardized protocol to ensure consistency across studies. Extracted variables included study characteristics, intervention types, outcomes, and implementation-related factors.

Data were synthesized using a narrative synthesis approach guided by PRISMA-ScR reporting principles and the evidence-mapping purpose of scoping reviews. Following data extraction, findings from the included studies were compared and organized according to recurring patterns in SSI prevention strategies, implementation issues, healthcare contexts, and reported outcomes. The synthesis was structured around the main areas of evidence identified across the included studies, allowing the review to map the breadth and nature of current SSI prevention evidence.

## 3 Findings

This scoping review synthesized evidence from 24 studies examining strategies for the prevention of surgical site infections (SSIs) across diverse healthcare settings. The 24 included studies represented a diverse body of evidence. In terms of study design, four studies were randomized controlled trials, 16 were observational or cohort-based studies, one was a model-based cost-effectiveness analysis, one used a mixed-methods design, and two were expert-based or guideline-focused papers. Geographically, the studies covered a wide range of settings, including Brazil, Saudi Arabia, Pakistan, Japan, Colombia, the United States, Spain, Switzerland, Canada, Sweden, China, Australia, Iran, France, and multinational Asia-Pacific and LMIC contexts. LMIC or middle-income settings were represented in eight studies, including studies from Brazil, Colombia, Pakistan, China, Iran, and the ChEETAh-related evidence from seven LMICs: Benin, Ghana, India, Mexico, Nigeria, Rwanda, and South Africa. The included studies examined antibiotic prophylaxis, antiseptic and decolonization strategies, negative pressure wound therapy, SSI prevention bundles, wound care practices, risk prediction models, AI-supported surveillance, and low-cost context-specific interventions. This range of designs, settings, and intervention types provided a broad basis for mapping current evidence on SSI prevention. A summary of the characteristics of the included studies is presented in Table 2.

**Table 2:** Data extraction.

Study title	Reference	Study design	Country setting	Population	Key finding	Prevalence to SSI prevention strategy
Evaluating antibiotic prophylaxis adherence: Implications for surgical site infections and wound care management	Bruna et al. (19)	Prospective observational cohort study	Brazil Teaching hospital, São Paulo, Brazil	Adults undergoing surgery with surgical antibiotic prophylaxis (*n* = 527; 78.7% completed follow-up, *n* = 415)	Adherence to the antibiotic protocol was critically low. - Prolonged antibiotic treatment was observed in 17.8% of participants. - Strict adherence could improve SSI outcomes and global antibiotic management.	Highlights the critical need for adherence to antibiotic prophylaxis protocols to reduce SSI incidence. Offers evidence for enhancing protocol compliance in healthcare settings.
Routine sterile glove and instrument change at the time of abdominal wound closure to prevent surgical site infection (ChEETAh)	Kachapila et al. (13)	Model-based cost-effectiveness analysis	Seven LMICs (specific data from India, Mexico, and Ghana) Healthcare providers in LMICs	Patients undergoing abdominal surgeries included in the ChEETAh trial. SSI rates were analyzed across seven LMICs, with costs modeled using KIWI data.	The intervention is cost-effective at all assumed thresholds ($0–$14,000). - Routine glove and instrument change before wound closure is feasible, cost-effective, and effective in LMICs.	Supports the adoption of a cost-effective, simple intervention to reduce SSI rates in resource-constrained settings.
Implementing a surgical site infection prevention bundle for emergency appendectomy	Jurt et al. (30)	Retrospective cohort	Switzerland Lausanne University Hospital (CHUV), Visceral Surgery Department	Adults undergoing emergency appendectomy	SSI rate decreased from 6.5% (pre-bundle) to 3.8% (post-bundle), with significant reduction in superficial incisional infections (*p* = 0.014). Compliance to bundle was 79.9%.	The study reports on a multi-item SSI prevention bundle with a demonstrated reduction in infections, highlighting a strong SSI prevention strategy for emergency appendectomy surgeries.
Implementation of a surgical site infection prevention bundle in gynecologic oncology patients	Ejaredar et al. (29)	Retrospective cohor	Canada Tom Baker Cancer Centre, Calgary, Alberta	Gynecologic oncology patients undergoing laparotomy	SSI rates decreased from 42.1% (pre-SSIPB) to 24.4% (post-SSIPB), with significant reductions in wound infections, UTIs, and intra-abdominal abscesses. Length of stay was shorter post-SSIPB.	The study highlights the effectiveness of a surgical site infection prevention bundle, showing a reduction in SSIs, UTIs, and other postoperative complications, demonstrating a successful SSI prevention strategy.
Impact of control measures including decolonization and hand hygiene for orthopaedic surgical site infection caused by MRSA at a Japanese tertiary-care hospital	Kawamura et al. (22)	Retrospective cohort	Japan Tertiary-care hospital	Orthopedic surgery patients	Reduction in MRSA SSI and outbreaks, with stronger adherence to hand hygiene and decolonization measures correlated with lower infection rates. Alcohol antiseptic use negatively correlated with SSI rates.	Hand hygiene and decolonization strategies were crucial in reducing MRSA-related SSIs.
Preventive strategies in paediatric cardiovascular surgery: impact on surgical site infections and beyond	Montoya et al. (27)	Cohort study	Colombia Paediatric cardiovascular surgery unit, Medellín	Children <12 years undergoing cardiovascular surgery for congenital heart disease (CHD)	SSI decreased from 9.5% in 2010 to 3.0% in 2021 (*p* = 0.030); antibiotic prophylaxis and VAC reduced SSI; no significant impact on other HAIs	Preventive strategies such as antibiotic prophylaxis based on weight and VAC were highly effective in reducing SSI.
Negative pressure wound therapy reduces surgical site infections	Benrashid et al. (26)	Cohort study	USA Vascular surgery unit, Durham, NC	504 patients with lower extremity or infrainguinal incisions	cNPWT significantly reduced SSI (9.8% vs. 19.0%, *p* < 0.01) and perioperative mortality (5.8% vs. 11.2%, *p* = 0.04). No significant differences in return to the operating room or readmissions.	Use of cNPWT as part of a care bundle effectively reduced SSI and perioperative mortality
Appropriateness of choice and duration of surgical antibiotic prophylaxis and the incidence of surgical site infections	Thabit et al. (21)	Prospective cohort study	Saudi Arabia King Fahad Armed Forces Hospital, Jeddah	98 adult patients undergoing abdominal or orthopedic surgery who received prophylactic antibiotics	72.9% of antibiotic choices were appropriate, and there was a significant association between appropriate antibiotic selection and lower SSI incidence (OR = 0.24, 95% CI 0.09–0.63). However, postoperative antibiotic duration did not fully comply with guidelines (compliance ranged from 0 to 13.6%).	Appropriate antibiotic selection was associated with a reduced SSI risk, but postoperative duration of antibiotic use did not align with guidelines.
Topical use of hyperoxygenated fatty acids decreases surgical site infection in patients following laparoscopic cholecystectomy	Bellón López de Antón Bueno et al. (21)	Randomized Controlled Trial	Spain General University Hospital of Elche, Alicante	103 patients undergoing laparoscopic cholecystectomy, with at least one risk factor for SSI or TSIH (BMI > 30, DM, age >65, COPD)	SSI rate was significantly lower in the HOFA group (3.8%) compared to the non-HOFA group (19.6%, *p* = 0.028). TSIH rates were similar between the two groups (3.8% vs. 2%).	Topical HOFA solution significantly reduced SSI rates, suggesting it as an effective preventive strategy for SSI after laparoscopic cholecystectomy.
Role of antibiotic prophylaxis on surgical site infection prevention in a low-risk population undergoing laparoscopic cholecystectomy	Ullah et al. (20)	Randomized Controlled Trial	Pakistan Pir Abdul Qadir Shah Jeelani Institute of Medical Sciences, Pakistan	240 low-risk patients scheduled for laparoscopic cholecystectomy	The SSI incidence was 3.98% in Group A (antibiotics) and 4.9% in Group B (no antibiotics), with no statistically significant difference (*p* = 0.584).	The study suggests that in low-risk patients undergoing laparoscopic cholecystectomy, prophylactic antibiotics do not significantly reduce SSI rates, indicating that their use may be unnecessary in this population.
Improving surgical site infection prevention in Asia-Pacific through appropriate surveillance programs	Russo et al. (3)	Expert Panel Discussion	Asia-Pacific Multinational (various APAC countries)	Expert opinions from 8 multidisciplinary specialists	Identified barriers include lack of standardized definitions, inconsistent reporting, limited resources, and cultural challenges. Recommendations for improving collaboration and harmonization across countries.	The study provides insights into improving SSI prevention strategies in the APAC region, emphasizing the importance of standardized surveillance systems.
Task-based training to prevent surgical site infection: A formative evaluation	Khan et al. (9)	Mixed-Methods (Design-based Research)	Pakistan Shifa International Hospital	Anesthesia and surgical trainees, registered nurses, technologists	The training had positive feedback, with participants valuing peer learning, video presentations, and practical examples of infection prevention.	The training program helped bridge the gap between knowledge and practice, with suggestions to further enhance the transfer of knowledge into practice.
Development and validation of a point-of-care clinical risk score to predict surgical site infection following open spinal fusion	Mueller et al. (34)	Quantitative (Retrospective Cohort, Logistic Regression)	USA Premier Healthcare Database, Open Spine Surgery	157,664 adult patients undergoing open spine surgery; 2,650 cases of SSI	The risk score is based on 16 predictors including patient demographics, comorbidities, surgical factors, and procedural details, with a C-statistic of 0.75 (reduced model) and 0.77 for validation.	The SSI risk score can be used to predict the risk of SSI in preoperative settings for patients undergoing open spinal surgery.
Impact of postoperative skin disinfection with chlorhexidine on bacterial colonization following shoulder arthroplasty surgery: a controlled randomized study	Markström et al. (25)	Randomized Controlled Trial (RCT)	Sweden County Hospital, Shoulder Arthroplasty Surgery	Adult patients undergoing elective shoulder arthroplasty surgery	The chlorhexidine group showed a higher prevalence of bacterial colonization with clinically relevant bacteria, though not statistically significant.	Postoperative skin disinfection does not appear to reduce bacterial colonization when compared with sodium chloride, challenging the common practice of using chlorhexidine for this purpose.
Construction and validation of nomogram to predict surgical site infection after hysterectomy: a retrospective study	Shao et al. (35)	Retrospective cohort study	China Tertiary Maternity and Child Specialist Hospital	Patients who underwent hysterectomy for gynecological malignancies or benign reproductive diseases resistant to medical treatment (2018–2023)	The nomogram demonstrated good calibration and discrimination, with high area under the ROC curve (AUC) and validation through clinical decision curve and impact curve analysis.	The nomogram provides a tool for early identification of high-risk patients, facilitating timely interventions to reduce SSIs post-hysterectomy.
Development of machine learning models for the detection of surgical site infections following total hip and knee arthroplasty: a multicenter cohort study	Wu et al. (14)	Retrospective Cohort Study	Canada Acute care hospitals in Calgary	Adult patients (≥18 years) undergoing primary elective total hip (THA) or knee (TKA) arthroplasty	The study demonstrates the feasibility of high-performance machine learning models for SSI detection using a combination of administrative and EMR data.	Machine learning models offer a novel approach to improving SSI detection accuracy and efficiency, aiding in early identification and management.
Strategies to Prevent Surgical Site Infections in Acute-Care Hospitals: 2022 Update	Michael Calderwood et al. (1)	Expert guidelines update	USA Acute-care hospitals	Healthcare providers and patients	Implementation of antimicrobial prophylaxis, decolonization, glucose control, and the use of antiseptics demonstrated effectiveness	60% of SSIs are preventable using evidence-based strategies.
intervention Strategies to Reduce Surgical Site Infection Rates in Patients Undergoing Rectal Cancer Surgery	Okada et al. (31)	Retrospective Control Study	Japan University Hospital	Patients undergoing rectal cancer surgery	Multivariate analysis identified factors such as laparoscopic surgery, absorbable sutures, and oral antibiotics as reducing SSI risk.	90% relative reduction in SSI rates achieved through combined interventions like MBP with oral antibiotics, laparoscopic surgery, and glove changes.
Effectiveness of Prophylactic Intranasal Photodynamic Disinfection Therapy and Chlorhexidine Gluconate Body Wipes for SSI Prophylaxis in Adult Spine Surgery	Moskven et al. (24)	Prospective Observational Interrupted Time-Series Study	Canada Level 1 Trauma Centre, Vancouver	Adult patients undergoing elective or emergent spine surgery	Demonstrated significant reductions in SSI rates, with cost savings of $2.48–$2.49 M annually, and no adverse events reported.	66% reduction in SSI incidence through preoperative nPDT–CHG decolonization bundle.
Wound care practices across two acute care settings: A comparative study	Gillespie et al. (10)	Prospective Comparative Observational Study	Australia Two metropolitan hospitals	154 nurses and 257 surgical patients	Low adherence to post-dressing hand hygiene (9.3%) and significant documentation differences.	Emphasizes standardizing wound care practices to reduce infection risks.
Operative site irrigation with povidone-iodine solution in spinal surgery for SSI prevention	Sigari and Abdolhoseinpour (28)	Randomized Controlled Trial	Iran Single hospital	936 patients undergoing spinal fusion surgery	PVI irrigation significantly reduced SSI rates without impairing fusion or wound healing.	PVI irrigation reduced SSI rates by 75% compared to saline irrigation.
Individual decontamination measures reduce by two the incidence of surgical site infections in spinal surgery	Bouyer et al. (23)	Bicentric Observational Study	France Two tertiary hospitals	5,314 spinal surgery patients	Significant reduction in SSI incidence with interventions including mupirocin and chlorhexidine decontamination.	Highlights the importance of decontamination measures for effective SSI prevention.
Machine Learning-Enhanced Surveillance for Surgical Site Infections in Patients Undergoing Colon Surgery	Celik et al. (32)	Retrospective study	USA, University of Massachusetts Memorial Medical Center	1,508 patient undergoing colon surgery (66 SSI cases,4.4%); study period January 2018–December 2023; SSI patients: mean age 61.1 years, 65% female; Non-SSI patients: mean age 58.5 years, 53.1% female	XG Boost achieved best performance (AUC0.788, precision 50%, recall 38%, Brier score 0.035). Top predictors: recovery duration (SHAP = 1.18), SSI keyword frequency (SHAP = 1.12), patient age (SHAP = 1.12), ASA score (SHAP = 0.94). SSI patients had longer hospital stays (8.1 vs. 6.3 days, *p* < 0.001), higher ASA score of 3 (79% vs 45.3%, *P* < 0.001), and elevated WBC counts (77% vs. 50.9%, *P* < 0.001). ML models can augment traditional SSI surveillance	Enhances SSI surveillance efficiency by prioritizing high-risk patients for infection control review. Automates surveillance to reduce burden on infection control practitioners. Integrates structured EHR data and unstructured clinical notes to improve detection accuracy. Supports scalable and efficient infection control practices. Enables early identification of patients at high risk for targeted intervention.
Evaluating the Performance of State-of-the-Art Artificial Intelligence Chat Bots Based on the WHO Global Guidelines for the Prevention of Surgical Site Infection	Tianyi Wang et al. (33)	Cross sectional study	China, Beijing Chao-Yang Hospital	300 responses from 4 AI chat bots (Claude 3.5 Sonnet, ChatGPT-4o, Gemini 1.5Pro, and one unnamed model) to 25queries derived from WHO global guidelines; evaluated by 4 senior surgeons from different subspecialties(orthopedic 31y, general 25y, thoracic 22y, urological 26y experience)	Overall performance scores (mean ± SD): Recommendations - Accuracy 4.03 ± 1.09, Consistency 4.07 ± 0.88, Harm 4.29 ± 1.01. Rationales - Harm 4.22 ± 0.97, Relevance 4.15 ± 0.83, Fabrication/Falsification 4.12 ± 1.02, Understanding/Reasoning 4.04 ± 0.92. Interrater agreement ranged from moderate to good (0.54–0.87). Claude 3.5 Sonnet and ChatGPT-4o were top performers. Gemini 1.5 Pro produced more inaccurate and harmful responses. AI chatbots showed acceptable performance for guideline-related responses.	Evaluates AI chatbots’ capability to deliver accurate SSI prevention recommendations aligned with WHO global guidelines. Demonstrates potential to alleviate surgeons’ information retrieval and decision-making burdens for SSI prevention. Identifies need for enhanced chatbot stability to prevent inaccurate responses that could lead to severe SSI consequences. Provides QUEST framework for evaluating healthcare AI applications in guideline adherence. Highlights AI’s potential to transform how clinicians’ access and use medical information for SSI prevention.

The findings were organized into five interrelated themes. The first theme, core clinical prevention strategies, includes interventions such as antibiotic prophylaxis, topical antisepsis and decolonization, and negative pressure wound therapy. The second theme, multicomponent approaches, focuses on prevention bundles that combine multiple practices to enhance consistency of care. The third theme, implementation barriers and facilitators, explores factors influencing adherence to preventive measures across different contexts. The fourth theme, artificial intelligence and data-driven approaches, reflects emerging strategies in risk prediction and automated surveillance. The final theme, context-specific interventions for low- and middle-income countries (LMICs), highlights cost-effective and adaptable approaches suited to resource-limited settings. Together, these themes demonstrate that SSI prevention requires the integration of clinical interventions, system-level implementation, and context-sensitive innovation.

The included studies contributed to five synthesis themes. Core Clinical Prevention Strategies were addressed in 13 studies; Multicomponent and System-Based Approaches in 6 studies; Implementation Barriers and Facilitators in 5 studies; Artificial Intelligence and Data-Driven Approaches in SSI Prevention in 4 studies; and Context-Specific and Resource-Sensitive Interventions in 3 studies. Some studies contributed to more than one theme because they addressed multiple dimensions of SSI prevention, such as intervention effectiveness, implementation issues, and contextual adaptation.

### 3.1 Core clinical prevention strategies

Across the included studies, several well-established clinical interventions consistently emerged as foundational to SSI prevention, including antibiotic prophylaxis, topical antisepsis and decolonization strategies, and negative pressure wound therapy (NPWT). These interventions operate through different mechanisms but share the common objective of reducing microbial contamination and improving perioperative outcomes.

Antibiotic prophylaxis remains a cornerstone of SSI prevention, with evidence demonstrating that appropriate selection, timing, and duration significantly reduce infection risk. For instance, Thabit et al. (6) reported that appropriate antibiotic selection was associated with a substantial reduction in SSI incidence (OR 0.24; 95% CI 0.09–0.63). However, adherence to recommended protocols remains inconsistent. Velozo et al. (19) identified non-compliance in 17.8% of cases, including prolonged postoperative antibiotic use, which may contribute to adverse outcomes and antimicrobial resistance. Similarly, Ullah et al. (20) found no significant difference in SSI rates between antibiotic and non-antibiotic groups in low-risk laparoscopic cholecystectomy patients, suggesting that inappropriate or unnecessary use may not yield additional benefit. These findings highlight that the effectiveness of antibiotic prophylaxis is closely tied to implementation fidelity rather than availability alone.

Across the included antibiotic prophylaxis studies, cephalosporin-based prophylaxis emerged as the most common pattern where antibiotic choice was specified. Cefazolin was the most recurrent named agent, particularly in orthopedic, upper abdominal, and laparoscopic cholecystectomy contexts, while cefoxitin and cefuroxime were also included in institutional prophylaxis protocols (6, 19, 20). In lower abdominal procedures, additional anaerobic coverage was evident, with metronidazole reported as the most common agent in this subgroup (6). Overall, the observed trend was toward procedure-specific, guideline-informed prophylaxis rather than broad-spectrum antibiotic use, with emphasis on appropriate agent selection, timing, redosing, duration, and selective omission of prophylaxis in low-risk procedures where benefit was limited (6, 19, 20).

Topical antiseptic and decolonization strategies also demonstrated considerable efficacy, particularly in high-risk populations. Bellón López de Antón Bueno et al. (21) reported significant reductions in SSI rates with the use of hyperoxygenated fatty acids in laparoscopic procedures, while Kawamura et al. (22) showed that mupirocin and chlorhexidine effectively reduced MRSA-related SSIs in orthopedic settings. However, their success depends heavily on proper application and adherence to protocols. Studies consistently identified gaps in provider knowledge and practice, which were mitigated through targeted training programs, including workshops and online modules (9, 23). In addition, patient education—such as preoperative counselling and culturally appropriate materials—was shown to improve compliance and hygiene practices (3, 23).

In adult spine surgery, Moskven et al. (24) reported that prophylactic intranasal photodynamic disinfection combined with chlorhexidine gluconate body wipes was associated with a reduction in SSI incidence and substantial cost savings. This suggests that decolonization strategies may be particularly useful when integrated into structured preoperative prevention protocols. However, findings related to chlorhexidine were not uniformly positive across all contexts. In a randomized study of postoperative skin disinfection following shoulder arthroplasty, Markström et al. (25) found no clear reduction in clinically relevant bacterial colonization compared with sodium chloride. This highlights that the effectiveness of antiseptic strategies may vary according to timing, procedure type, target organism, and mode of application.

Negative pressure wound therapy emerged as a valuable adjunctive strategy, particularly for high-risk surgical populations. Benrashid et al. (26) reported a reduction in SSI rates from 19.0 to 9.8%, alongside decreased perioperative mortality. Similar benefits were observed in pediatric cardiovascular settings, where preventive strategies incorporating NPWT contributed to improved outcomes (27). Despite its effectiveness, the widespread adoption of NPWT remains constrained by cost and the need for specialized training, particularly in resource-limited settings (19). Adaptations such as local manufacturing, targeted training, and telemedicine support have been proposed to improve accessibility (3, 9). Additional intraoperative antiseptic innovations, such as operative site irrigation with povidone-iodine solution during spinal surgery, demonstrated potential in reducing SSI risk by directly lowering wound contamination while maintaining safety (28), further reinforcing the value of localized antiseptic strategies.

Taken together, these findings demonstrate that while core clinical interventions are strongly supported by evidence, their effectiveness is highly dependent on consistent and context-sensitive implementation.

### 3.2 Multicomponent and system-based approaches

Beyond individual interventions, the review highlights the effectiveness of multicomponent strategies, particularly SSI prevention bundles, which integrate several evidence-based practices across the perioperative pathway. Unlike single interventions, these bundles are designed to address multiple sources of infection risk simultaneously, thereby reducing variability in clinical practice and improving overall adherence to preventive measures.

Across the included studies, bundles typically combined elements such as appropriate antibiotic prophylaxis, preoperative skin preparation (e.g., chlorhexidine bathing), maintenance of intraoperative sterility, wound protection measures, and postoperative monitoring. In some cases, they also incorporated intraoperative practices such as glove and instrument changes before wound closure, temperature control, and optimization of operating room protocols. These combinations reflect a systems-based approach in which no single measure is sufficient, but collectively they create a more robust barrier against infection.

Empirical evidence from the included studies supports the effectiveness of such approaches. For example, Ejaredar et al. (29) implemented a comprehensive SSI prevention bundle in gynecologic oncology patients that included antibiotic optimization, antiseptic preparation, and perioperative care standardization, resulting in a reduction in SSI rates from 42.1 to 24.4%. Similarly, Jurt et al. (30) demonstrated that a structured bundle applied in emergency appendectomy—comprising timely antibiotic administration, improved surgical site preparation, and standardized intraoperative practices—reduced SSI incidence from 6.5 to 3.8%. Evidence from rectal cancer surgery further supports the value of procedure-specific multicomponent strategies; Okada et al. (31) reported a marked reduction in SSI rates following combined interventions that included mechanical bowel preparation with oral antibiotics, laparoscopic surgery, absorbable sutures, and glove changes. In LMIC contexts, the ChEETAh trial further highlighted the effectiveness of a simplified bundle approach, where routine glove and instrument changes prior to wound closure significantly reduced SSI rates across multiple countries (13).

The effectiveness of these bundles appears to lie in their ability to operationalize guidelines into routine practice. By structuring care into clearly defined steps, bundles reduce reliance on individual clinician discretion and minimize the risk of omitted or delayed interventions. However, their success is closely linked to implementation capacity. In many LMIC settings, limited access to sterile supplies, inadequate infrastructure, and workforce constraints hinders full adoption (3). Additionally, high workloads and insufficient training can reduce adherence to bundle components (9).

To address these challenges, several studies reported the effectiveness of adapted or simplified bundles tailored to local resource constraints. For instance, the study by Khan et al. (9) highlighted the value of task-based training in strengthening infection-prevention learning and translating evidence-based practices into clinical routines, particularly in settings where gaps between knowledge and practice may affect adherence to SSI prevention measures.

Overall, these findings indicate that the success of multicomponent strategies depends not only on the inclusion of evidence-based elements, but also on their adaptability to local contexts and the presence of supporting systems that ensure consistent implementation.

### 3.3 Implementation barriers and facilitators

A consistent finding across the included studies is that the primary challenge in SSI prevention lies not in the absence of effective interventions, but in the difficulty of implementing them reliably in routine clinical practice. Barriers were multifactorial, encompassing structural, organizational, and behavioral dimensions. Commonly reported challenges included limited availability of resources, inadequate training of healthcare personnel, lack of access to updated clinical guidelines, and inconsistencies in adherence to established protocols across surgical settings.

In low- and middle-income countries (LMICs), these barriers were particularly pronounced due to infrastructural constraints, shortages of essential supplies, and underdeveloped infection prevention systems (3). For example, limited access to sterile equipment, inconsistent availability of antibiotics, and overcrowded surgical environments were reported to hinder compliance with standard infection control practices. However, implementation challenges were not confined to resource-limited settings. Even in high-income contexts, issues related to workflow integration, staff workload, and variability in clinical practice were evident. Delayed antibiotic administration and inappropriate postoperative antibiotic use remained persistent concerns, reducing the effectiveness of otherwise well-established preventive measures (6, 19). These findings indicate that the presence of resources alone does not guarantee optimal implementation.

Behavioral and knowledge-related factors also played a significant role. Several studies highlighted gaps in healthcare providers’ understanding of SSI prevention protocols, particularly regarding timing, dosage, and duration of antibiotic prophylaxis (6, 19). In addition, variations in clinical judgement and reliance on habitual practices contributed to inconsistencies in care delivery. High workload and time constraints further limited the ability of healthcare professionals to adhere strictly to all recommended preventive steps (20).

Conversely, a range of facilitators were identified that supported improved implementation. Training programs, particularly those combining theoretical instruction with practical application, were consistently associated with enhanced adherence to SSI prevention practices (9). Educational interventions tailored to local contexts were especially effective in addressing knowledge gaps and improving clinical competence. Furthermore, the introduction of audit and feedback mechanisms enabled continuous monitoring of compliance and provided healthcare teams with actionable insights to improve practice. Institutional policies and leadership support were also critical, as they reinforced accountability and standardization across departments (3).

Overall, these findings highlight that SSI prevention is fundamentally an implementation challenge, requiring coordinated efforts across clinical practice, organizational systems, and policy frameworks to ensure consistent and sustained application of evidence-based interventions.

### 3.4 Artificial intelligence and data-driven approaches in SSI prevention

Recent evidence demonstrates a clear shift toward artificial intelligence (AI) and data-driven approaches as emerging adjuncts in surgical site infection (SSI) prevention. Unlike conventional strategies that rely on standardized protocols and retrospective surveillance, these approaches enable proactive risk identification, automated monitoring, and enhanced decision support. The included AI-related studies represented two distinct applications: machine learning and predictive analytics for SSI detection, surveillance, and risk prediction, and large language models (LLMs) or chatbots for guideline-based knowledge support. Collectively, they reflect a transition from reactive infection control toward predictive, data-informed prevention systems (32, 33).

A consistent finding across studies is the effectiveness of machine learning in improving SSI detection and surveillance. For example, Celik et al. (32) developed models integrating structured electronic health record data with natural language processing (NLP) features, achieving strong predictive performance (AUC = 0.788) and enabling prioritization of high-risk patients in clinical workflows. Similarly, a large multicenter cohort study by Wu et al. (14) reported even higher performance when combining administrative data with free-text clinical notes, with XGBoost models achieving an AUC of 0.906 and F1 score of 0.79. Importantly, this study demonstrated that incorporating unstructured nursing notes significantly improves detection accuracy compared to structured data alone, highlighting the value of multimodal data integration in SSI surveillance (14).

These findings collectively reinforce that AI-driven surveillance systems are particularly valuable in addressing one of the most persistent challenges in SSI prevention—labor-intensive and often incomplete manual chart review. By automating case detection and risk stratification, such models can reduce workload while improving sensitivity and consistency of surveillance. However, across both studies, AI performance remains subject to trade-offs between sensitivity and precision, confirming that these systems function best as decision-support tools rather than autonomous diagnostic solutions (14, 32).

Beyond surveillance, predictive modelling using statistical–AI hybrid approaches is emerging as a practical tool for early intervention. Mueller et al. (34) developed and validated a point-of-care clinical risk score to predict SSI following open spinal fusion, using routinely available preoperative and procedural variables to support individualized risk stratification. Similarly, Shao et al. (35) developed a nomogram-based model for SSI following hysterectomy, identifying key predictors such as elevated BMI, hypoproteinemia, prolonged antibiotic use, prior abdominal surgery, extended hospital stays, and malignant pathology. The model demonstrated strong discriminative ability (AUC up to 0.935) and clinical utility through decision curve analysis, supporting its role in personalized risk assessment and targeted prevention strategies (35). Compared with more complex machine learning models, such tools offer greater interpretability and may facilitate integration into routine clinical practice. In parallel, large language models (LLMs) represent a distinct but complementary AI application focused on knowledge translation. Unlike machine learning models that detect SSI cases or estimate patient risk, chatbot-based systems are designed to support access to guideline-based information and clinical reasoning. AI chatbots can provide guideline-based recommendations for SSI prevention with generally acceptable performance, particularly in relevance, reasoning, and safety domains (33). These systems offer a novel mechanism for clinicians to access and interpret rapidly evolving SSI guidelines, potentially addressing challenges related to information overload and time constraints in clinical practice (33).

However, important limitations persist across all AI applications. Chatbot responses showed only moderate performance in accuracy, clarity, and comprehensiveness, with instances of inconsistency and potentially harmful outputs, particularly in complex or ambiguous clinical scenarios (33). Similarly, predictive and surveillance models depend heavily on robust electronic health record infrastructure and high-quality data inputs, which may not be universally available (14, 32). Thus, the limitations differ by AI type: surveillance and prediction models require reliable electronic health record infrastructure, external validation, and workflow integration, whereas chatbot-based tools require careful evaluation of accuracy, consistency, transparency, and potential clinical harm. These findings highlight a critical boundary: AI systems perform most reliably when reinforcing established evidence but remain less dependable in uncertain or context-specific situations.

Taken together, the four studies illustrate two primary contributions of AI to SSI prevention: first enhancing surveillance and early detection through machine learning and predictive modelling, and second improving access to and interpretation of guideline-based knowledge through conversational AI systems. Importantly, these approaches directly address key implementation gaps identified in the broader literature, including delayed identification of high-risk patients, variability in surveillance practices, and challenges in maintaining adherence to evolving guidelines.

Despite these advances, the integration of AI into SSI prevention remains uneven and context-dependent. Effective implementation requires not only technological capability but also alignment with clinical workflows, data governance, and ongoing human oversight. Furthermore, reliance on advanced digital infrastructure raises concerns about scalability and equity, particularly in low-resource settings where SSI burden is often highest.

Overall, AI and data-driven approaches represent a promising yet still evolving domain within SSI prevention. Current evidence positions these technologies as valuable adjuncts that enhance efficiency, support clinical decision-making, and strengthen implementation of existing prevention strategies, rather than replacing them. Future research should prioritize real-world implementation, cross-setting validation, and the development of context-sensitive models capable of operating reliably across diverse healthcare environments.

### 3.5 Context-specific and resource-sensitive interventions

The importance of context-specific approaches is particularly evident in studies conducted in low- and middle-income countries (LMICs), where resource constraints necessitate adaptation of standard SSI prevention strategies. Across the included studies, LMIC evidence was represented by research conducted in Benin, Ghana, India, Mexico, Nigeria, Rwanda, South Africa, and Pakistan. Cost-effective and feasible interventions were shown to produce meaningful improvements in SSI outcomes, even in settings with limited infrastructure and access to advanced technologies. These findings highlight that effective SSI prevention does not depend solely on high-cost or technologically advanced interventions, but rather on the ability to implement practical measures that align with local conditions.

A key example is provided by the ChEETAh trial (13), which was conducted across seven LMICs, namely Benin, Ghana, India, Mexico, Nigeria, Rwanda, and South Africa. The trial demonstrated that routine glove and instrument changes before wound closure significantly reduced SSI rates across multiple countries. This intervention, while simple and low-cost, directly addressed a critical point of contamination during surgery and was shown to be both feasible and scalable across diverse healthcare environments. Its effectiveness underscores the potential of targeted, evidence-based practices that can be implemented without requiring substantial financial investment or specialized equipment. A related economic analysis further supported the cost-effectiveness of this intervention using pooled trial outcomes and costing data from India, with additional analyses based on Mexico and Ghana (13).

Evidence from Pakistan also highlighted the importance of aligning prevention strategies with patient risk and antimicrobial stewardship considerations. In low-risk patients undergoing laparoscopic cholecystectomy, routine antibiotic prophylaxis did not significantly reduce SSI rates, suggesting that context-sensitive prevention should involve not only introducing low-cost interventions but also avoiding unnecessary practices where clinical benefit is limited. Together, these findings indicate that SSI prevention in LMICs requires practical, scalable, and resource-sensitive strategies that can be standardized within local surgical workflows.

In addition to specific interventions, broader system-level factors were identified as essential to successful implementation in LMIC contexts. International collaboration played a significant role in supporting infection prevention efforts, particularly through the provision of resources, technical expertise, and training initiatives (3). Partnerships between local healthcare institutions, governmental bodies, and international organizations facilitated the adaptation of global guidelines to local realities, ensuring that recommended practices were both applicable and sustainable.

Training and capacity-building initiatives were also critical facilitators. Educational programs tailored to the needs of healthcare workers in resource-limited settings improved knowledge, skills, and adherence to infection prevention protocols (9). These initiatives often focused on reinforcing fundamental practices such as aseptic technique, appropriate antibiotic use, and perioperative care, which are essential components of SSI prevention but may be inconsistently applied in under-resourced environments.

Community engagement further contributed to the success of these interventions by enhancing patient awareness and participation in infection prevention measures. Culturally appropriate education and communication strategies were important in promoting adherence to hygiene practices and postoperative care instructions, particularly in settings where health literacy may vary.

Overall, these findings emphasize that SSI prevention strategies must be tailored to local contexts, taking into account available resources, infrastructure, and workforce capacity. In LMIC settings, practical, scalable interventions supported by training, collaboration, and institutional commitment can achieve substantial improvements in surgical outcomes, reinforcing the importance of context-sensitive approaches in global infection prevention efforts.

## 4 Discussion

This scoping review provides a comprehensive synthesis of current evidence on surgical site infection (SSI) prevention, demonstrating that effective infection control extends beyond the presence of evidence-based interventions to encompass how these interventions are implemented, integrated, and adapted across healthcare contexts. The findings collectively suggest that SSI prevention should be understood as a multi-layered process in which clinical practices, system-level organization, and contextual factors interact to determine outcomes.

A key insight emerging from this review is the central role of implementation in shaping the effectiveness of established preventive strategies. While interventions such as antibiotic prophylaxis, antiseptic preparation, and advanced wound therapies are supported by strong evidence, their impact is highly dependent on consistent and appropriate application in clinical practice. Variability in adherence, influenced by factors such as workflow pressures, knowledge gaps, and organizational constraints, continues to limit the real-world effectiveness of these interventions (3, 7, 19). This reinforces a broader understanding that the gap between evidence and practice remains a critical challenge in infection prevention, even in settings where resources are available.

The findings also highlight the importance of system-based approaches in addressing this gap. Multicomponent strategies, particularly prevention bundles, demonstrate that structuring care processes can significantly improve consistency and reduce variability in clinical practice. Their effectiveness lies not only in the combination of interventions, but in their ability to standardize care delivery and support coordinated implementation (29, 30). This suggests that SSI prevention is as much an organizational challenge as it is a clinical one, requiring systems that facilitate reliability, accountability, and continuous monitoring.

At the same time, the review underscores the significance of contextual variation, particularly in low- and middle-income countries (LMICs). Resource constraints, infrastructural limitations, and workforce challenges shape both the feasibility and effectiveness of SSI prevention strategies. However, the success of simplified and resource-sensitive interventions indicates that meaningful improvements can be achieved without reliance on high-cost technologies. Instead, interventions that are feasible, scalable, and aligned with local conditions appear to be more sustainable and impactful (3, 9, 13). This finding supports the need for greater emphasis on context-adapted approaches in global infection prevention efforts.

The findings of this review highlight the increasing integration of artificial intelligence (AI) and data-driven approaches in surgical site infection (SSI) prevention and surveillance. Emerging evidence demonstrates that machine learning–based models can enhance early risk prediction and streamline surveillance processes by leveraging both structured and unstructured clinical data, thereby reducing reliance on manual chart review and improving efficiency (32). In parallel, other studies have shown that predictive analytics and algorithm-based systems can support real-time clinical decision-making and optimize infection control strategies, particularly in high-risk surgical populations (14, 35).

Additionally, the use of AI-powered tools such as clinical decision support systems and automated surveillance platforms has been associated with improved detection accuracy and workflow integration, enabling more proactive and targeted interventions (34). More recently, the application of AI chatbots has introduced a novel dimension to SSI prevention by facilitating access to guideline-based recommendations; however, concerns regarding variability in accuracy, consistency, and clinical reliability persist (33).

Collectively, these findings reflect a broader shift toward intelligent, data-driven healthcare systems that augment traditional infection prevention practices. Nevertheless, despite their potential, AI-based tools remain dependent on data quality, contextual adaptation, and human oversight. Therefore, their role should be viewed as complementary rather than substitutive, reinforcing clinical judgment and supporting adherence to evidence-based SSI prevention strategies.

Taken together, the findings suggest a conceptual shift in SSI prevention from a predominantly protocol-driven model toward a more integrated and adaptive approach. Traditional interventions remain foundational, but their effectiveness is increasingly shaped by implementation systems and contextual realities. Emerging technologies further extend this model by introducing data-informed decision-making, although their role is still evolving.

From a practical perspective, these insights highlight the importance of strengthening implementation capacity through training, monitoring systems, and institutional support. Healthcare systems should prioritize interventions that are not only evidence-based but also feasible within their specific context. Future research should focus on evaluating the long-term effectiveness and scalability of both established and emerging strategies, particularly in resource-limited settings where the burden of SSI remains high.

Overall, this review contributes to the literature by framing SSI prevention as a dynamic and multi-dimensional process that requires alignment between clinical evidence, system-level implementation, and contextual adaptation. Addressing these interconnected factors is essential for achieving sustained reductions in SSI rates and improving surgical outcomes globally.

This review contributes to the existing literature by integrating clinical, implementation, and technological perspectives into a unified analytical framework. While previous studies have examined individual components of SSI prevention, this synthesis highlights the interaction between intervention effectiveness, implementation capacity, and contextual constraints across diverse healthcare settings.

This review has several limitations. The restriction to English-language publications may have excluded relevant studies, particularly from non-English-speaking regions. In addition, the selection of Scopus, Web of Science, and ScienceDirect as the primary information sources may have influenced the scope of retrieved studies, and relevant records indexed only in other biomedical databases may not have been captured. Moreover, the heterogeneity of study designs and outcomes limits direct comparability across studies. As a scoping review, this study did not aim to assess the effectiveness of interventions quantitatively, and findings should therefore be interpreted as indicative of trends rather than definitive conclusions. Despite these limitations, this review provides a broad and analytically integrated overview of current SSI prevention strategies, implementation challenges, and emerging innovations, offering valuable guidance for future research, policy development, and clinical practice.

## 5 Conclusion

This scoping review synthesizes current evidence on surgical site infection (SSI) prevention, highlighting that effective infection control extends beyond individual clinical interventions to include system-level implementation and contextual adaptation. While established strategies such as antibiotic prophylaxis, topical antisepsis, and negative pressure wound therapy remain essential, their impact depends on consistent application and integration within routine practice. Multicomponent approaches, particularly prevention bundles, strengthen outcomes by improving reliability and reducing variability in care delivery. The findings further emphasize the importance of context-sensitive strategies in low- and middle-income countries, where feasible and scalable interventions can achieve substantial improvements. Emerging technologies, including artificial intelligence, offer additional opportunities to enhance prevention through improved risk prediction and surveillance. Ultimately, advancing SSI prevention requires coordinated alignment between evidence-based practice, implementation systems, and context-aware innovation to achieve sustainable improvements in surgical outcomes.

## Data Availability

The original contributions presented in the study are included in the article/Supplementary material, further inquiries can be directed to the corresponding author/s.
