# Supplementary material for: Surgical site infection prevention: a scoping review of strategies, implementation barriers, and emerging approaches
Source: Front Public Health. 2026 Jul 30;14:1884446. doi: 10.3389/fpubh.2026.1884446 (PMC13469273; doi:10.3389/fpubh.2026.1884446)
Supplement: Supplementary file 1 [file Supplementary_file_1.DOCX]

**Supplementary File A. Database-Specific Search Strings**

| **Database** | **Search string** |
| --- | --- |
| Web of Science | ("surgical site infection" OR SSI OR "postoperative wound infection") AND (prevention OR control OR intervention* OR strateg*) AND ("antibiotic prophylaxis" OR antisepsis OR decolonization OR "negative pressure wound therapy" OR NPWT OR bundle* OR checklist) AND (hospital* OR surgical OR perioperative) |
| Scopus | ("surgical site infection" OR SSI OR "postoperative wound infection") AND (prevention OR control OR intervention* OR strateg*) AND ("antibiotic prophylaxis" OR antisepsis OR decolonization OR "negative pressure wound therapy" OR NPWT OR bundle* OR checklist) AND (hospital* OR surgical OR perioperative) |
| ScienceDirect | ("surgical site infection" OR SSI) AND (prevention OR control) AND ("antibiotic prophylaxis" OR antisepsis OR NPWT OR bundle) |
